# Changes in body composition during the macrocycle of professional football players in relation to sports nutrition knowledge

**DOI:** 10.3389/fnut.2022.981894

**Published:** 2022-11-29

**Authors:** Wiktoria Staśkiewicz, Elzbieta Grochowska-Niedworok, Grzegorz Zydek, Agnieszka Białek-Dratwa, Mateusz Grajek, Sylwia Jaruga-Sȩkowska, Oskar Kowalski, Marek Kardas

**Affiliations:** ^1^Department of Food Technology, Quality Evaluation, and Dietetics, Faculty of Health Sciences in Bytom, Medical University of Silesia in Katowice, Zabrze, Poland; ^2^Department of Health Sciences and Physical Culture, University of Applied Sciences in Nysa, Nysa, Poland; ^3^Department of Sport Nutrition, Jerzy Kukuczka Academy of Physical Education in Katowice, Katowice, Poland; ^4^Department of Human Nutrition and Dietetics, Faculty of Health Sciences in Bytom, Medical University of Silesia in Katowice, Zabrze, Poland; ^5^Department of Public Health, Public Health Policy, Faculty of Health Sciences in Bytom, Medical University of Silesia in Katowice, Bytom, Poland; ^6^Department of Health Promotion, Faculty of Health Sciences in Bytom, Medical University of Silesia in Katowice, Bytom, Poland

**Keywords:** body composition, nutrition knowledge, football, professional athletes, anthropometry

## Abstract

Professional football players are obligated to meet the physical demands and maintain the best possible performance throughout the whole macrocycle. It is important to assess the players' nutrition knowledge, identify areas that require increased nutrition awareness and identify the impact of knowledge on changes in body composition as this can affect the players' health and performance. This study aimed to assess changes in the body composition of professional football players during the macrocycle of the spring round of the football championship and to identify the correlation between nutrition knowledge and maintaining body composition. The study included 38 football players. The players' body compositions were analyzed 6 times during the macrocycle consisting of preparatory, competitive, and transition periods using the Direct Segmental Multi-Frequency Bioelectrical Impedance Analysis method. Athletes completed the Nutrition for Sport Knowledge Questionnaire to assess their nutrition knowledge. During the preparatory period, a statistically significant negative correlation was demonstrated between the players' knowledge about the subsections of micronutrients in the diet and the dispersion of the adipose percentage tissue content (*r* = −0.36, *p* = 0.03). In the competitive period, there was a statistically significant negative correlation between the players' knowledge of sports nutrition and the dispersion of lean body mass (*r* = −0.46, *p* = 0.004), and skeletal muscle mass (*r* = −0.36, *p* = 0.03). During the transition period, a statistically significant negative correlation between the players' knowledge of weight control and the dispersion of body mass (*r* = −0.47, *p* = 0.00) and BMI values (*r* = −0.48, *p* = 0.00) was identified. The player's knowledge about the subsection of macronutrients significantly negatively correlated with the dispersion of skeletal muscle mass content (*r* = −0.33, *p* = 0.05). Nutrition knowledge has an impact on the stability of body composition during all analyzed periods: preparatory, competitive, and transition periods.

## Introduction

Football^1^ players are required to meet physical demands and maintain top physical condition throughout the whole macrocycle. For this reason, constant assessment of players' physical condition plays a key role in the success of any team (1). Anthropometric characteristics and body composition were proven to have a significant impact on players' performance (2–4), and studies report changes in them over the course of the football training periods (5, 6).

In football, depending on a calendar of competitions, the preparation of players occurs in one-cycle, two-cycle, or three-cycle models (7). The two-cycle model of the training process takes into consideration competition in a spring and fall system. One round constitutes a 6-month macrocycle, referred to as a round. In one round, we distinguish three periods: preparatory, competitive, and transition periods, individual periods of the macrocycle are successive stages of managing the development of the sport form (7, 8). The preparatory period is designed to develop athletic prowess in preparation for the competitive period (9). Training during this period is geared toward rebuilding athletes' physical fitness after the transition period (10–12). The competitive period is difficult to plan because training loads should be adjusted to maximize physiological adaptations and, at the same time, to avoid overtraining and injuries of players. This period consists in maximizing the result of the sport during matches based on the players' performance and skills dispositions obtained during the preparatory period (8, 13). The transition period is characterized by a complete cessation or significant reduction of training. Athletes may be engaged in recreational sporting activities or voluntary, non-periodic training during this time (14). Cessation of physical activity, the reduction in training, and the fitness level of the players will modulate the kinetics of changes in body weight and physiological functions (14–17). This period should be regarded by athletes as an opportunity to recover before the next season (18). There are few scientific research reports on how the body composition is shaped during periods in the football season (6, 19–22). It is extremely important to evaluate body composition during the entire macrocycle (preparatory, competitive, and transition periods) due to the differences in training intensity, care of the coaching staff, amount of match play, and amount of free time during each period. One training macrocycle (spring round) was included in the study to analyse each period (13, 20, 22).

One of the most common causes of inadequate diets amongst athletes is poor nutrition knowledge derived from inappropriate sources (23). To improve nutrition knowledge, it is required to know the areas that are characterized by the lowest nutritional awareness in the group of athletes and to identify the correlation between them and body weight composition (24). Poor knowledge of nutrition among professional football players can lead to bad eating behaviors. This results in an energy imbalance, weight gain or loss, decreased exercise performance, or increased risk of injury and illness (25, 26). Therefore, adequate nutrition knowledge and optimal body composition are important for the athletic performance of professional football players (26).

This study aimed to assess the body composition of professional football players during the preparatory, competitive, and transition periods and to identify the influence of their nutrition knowledge on body composition modification.

## Materials and methods

### Study design

The research was conducted during the spring round of the PKO BP Ekstraklasa football championship (the highest league of football competitions in Poland) between 7 January 2021 and 23 July 2021. Participating athletes were players of two Silesian football clubs belonging to PKO BP Ekstraklasa 2020/2021 (the highest league of football competitions in Poland). The study was conducted with the approval of the Bioethics Committee of the Silesian Medical University in Katowice (PCN/0022/KB/68/I/20).

Body composition analyses were conducted in three periods of the spring round (preparatory, competitive, and transition periods) of the PKO BP Ekstraklasa 2020/2021 football championship. The days on which the analyses were conducted were planned to take into account league and cup games, ensuring adequate time for post-match regeneration.

All players' nutritional knowledge of sports nutrition was assessed in the study. Due to the presence of athletes in the study group who did not speak Polish, nutritional consultations were conducted in Polish or English.

**Figure 1:**
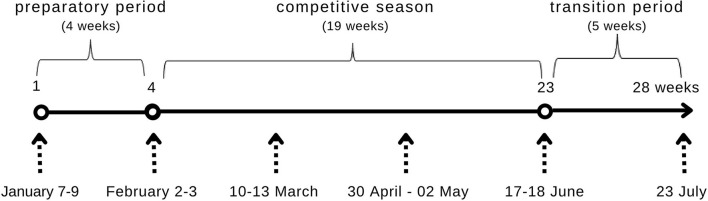
Procedure for the anthropometric measurements.

The next step was to analyse the correlation of differences between the maximum and minimum measurements of body composition in the studied period and nutritional awareness. The contribution of nutrition knowledge to the stability of body composition (measured by the difference between maximum and minimum measurements) during the three periods was assessed (Figure 1). The following were taken into account:

- preparatory period (1–2 measurements)
- competitive period (2–5 measurements)
- transition period (5–6 measurements).

### Participants

The study included 58 athletes aged 20–31. The participants in the study were of different nationalities (36 Polish, six Slovakian, three Spanish, three Portuguese, two Greek, two Slovenian, one Czech, one Austrian, one Danish, one Hungarian, one Ghanaian, and one Gambian). The classification was made in terms of function on the field: forward, midfielder, defender, and goalkeeper. Considering the inclusion and exclusion criteria, 228 complete body composition measurements were finally obtained from 38 athletes.

Inclusion criteria in the study were defined as follows: professional training of football in the clubs included in the study, giving consent to participate in the study, and no injury requiring long-term treatment that would exclude players from training and playing matches during the period of the study, i.e., 7 months.

Exclusion criteria for the study included: not speaking Polish or English at a communicative level, exclusion of players from training and playing matches for at least consecutive 14 days due to injury, illness, quarantine, or isolation related to COVID-19 pandemic, transfer to another football club during the study period, i.e., 7 January 2021 to 23 July 2021, absence from at least one of the six measurements for a reason other than the above.

The study included first-team players as well as reserve players. However, in both clubs, players who did not play in the championship match were obliged to participate in the additional match to maintain the match rhythm. Therefore, the players' game loads may be comparable.

### BMI

Height measurements were taken immediately before the first body composition measurement, while body weight was measured during the body composition analysis.

Height (cm) and body mass (kg) were measured to the nearest 0.1 cm (SECA 756, Seca GmbH & Co. KG, Hamburg, Deutschland) and 0.1 kg (InBody 770, InBody USA, California, USA), respectively, with the subject wearing underwear and no shoes. Body mass index (BMI) was calculated as body mass (kg) divided by height (m) squared. The results formed the basis for assessing height–weight ratios in relation to the standards of the European population and WHO (World Health Organization) recommendations (27).

### Body composition analysis

Body composition was assessed using a DSM-BIA (Direct Segmental Multi-Frequency Bioelectrical Impedance Analysis) (InBody 770, InBody USA, California, USA). The DSM-BIA technique is based on assumption that the human body is composed of five interconnecting cylinders and takes direct impedance measurements from the various body compartments. A tetrapolar eight-point tactile electrode system is used, which separately measures the impedance of the subject's torso, arms, and legs at six different frequencies (1, 5, 50, 250, 500, and 1,000 kHz) for each of the body segment. The analyser allows for obtaining a complete body composition in about 60 s. The device works with the use of a current of 80 uA (28, 29). At the molecular level, total body water (TBW) consists of fat mass (FM) and fat-free mass (FFM). Fat-free mass is in turn divided into compartments: lean body mass (LBM) and bone mineral mass (BMC). Lean body mass (LBM) is the sum of body water, total protein, carbohydrates, fat-free lipids, and soft tissue, excluding fat mass and bone mineral mass.

Body composition parameters were obtained using the Lookin'Body Software version 120.3.0.0.6. The measurements were conducted according to a standard protocol as recommended by the device manufacturer. Before each testing session, the analyser was checked with a calibration circuit of known impedance (resistance = 500.0 Ω; reactance = 0.1 Ω; 0.9% error). The participants were on an empty stomach and did not consume alcohol or caffeine for at least 24 h before the examination. The measurement was performed at a regular time, after defecation, at least 24 h after the end of intensive physical activity, without shoes and socks, in underwear, with clean and dried feet and hands without applied cream and lotion. The participants had to step on the foot electrodes barefoot and maintain an evenly distributed weight on the measurement platform while holding a pair of electrodes fixed to the display unit. Then, the participants extended their arms in front of the chest, maintaining a steady position until the measurements were completed.

Recently, BIA technology has developed significantly, allowing more accurate estimation of TBW, FFM, LBM, FM, and other results. Dual-energy X-ray absorptiometry (DEXA) is considered the reference method for assessing body composition, but there are some limitations. DXA is not portable, is expensive, and often requires training by a licensed technician due to its low radiation exposure. Bioelectrical impedance analysis has evolved to use multiple frequencies and impedance measurements to improve the validity and reliability of body composition estimates. Compared to other methods, BIA is relatively simple, quick, and non-invasive. The DSM-BIA method used in the InBody 770 compared to the reference DEXA method is valid and reliable in populations of healthy athletes who are clinically and physically inactive (30–33).

### Nutrition knowledge

To assess the nutrition knowledge of the study group, a survey method was applied using the Nutrition for Sport Knowledge Questionnaire (NSKQ) (34, 35). Assessment of the nutrition knowledge of the study group was carried out using the original version of the questionnaire in English and the Polish version. The NSKQ questionnaire developed by Trakman et al. was validated using an eight-item methodology. The researchers performed a reliability assessment using PerSepIndex, a summary statistic created by RUMM 2030, which is analogous to Cronbach's α. Both PerSepIndex and Cronbach's α is based on multiple split-half reliability assessments; ≥0.7 is an accepted measure of internal reliability (35). The authors of the questionnaire gave their consent for its use in the study. For this study, a pilot study was conducted using the Polish version of the questionnaire on a group of 30 people. The pilot study was conducted to validate the Polish version of the questionnaire and to check the accuracy and acceptability of the questions included in it. The original version of the survey instrument was translated into Polish by two independent English translators. For both, their native language was Polish. Based on the two translations, a Polish-language version of the NSKQ questionnaire was created, then all the sentences of the translation were discussed until the opinions were consistent. These procedures resulted in a version that met the condition of semantic consistency for each response. Two back-translations were then created by other independent translators. These were reviewed for consistency along with the original version by a person whose native language is English. The next step was to adapt the questionnaire, which maintained consistency in terms of graphics (font, text size, distribution of questions and answers, and amount of text per page) with the original version.

Cronbach's α coefficient for sample normalization was 0.83, indicating the high reliability of the questions. The pilot study allowed to validate the questions included in the Polish language questionnaire. The PerSepIndex coefficient for the actual part of the study was estimated at 0.8, which is the same value as the obtained Cronbach's α coefficient in the pilot study (34, 35). The questionnaire contained 87 questions divided into six subsections: weight control (*n* = 12), macronutrients (*n* = 30), micronutrients (*n* = 13), sports nutrition (*n* = 12), supplementation (*n* = 12), and alcohol (*n* = 8). Each section in the questionnaire used is one-dimensional; therefore, individual sections can be used independently to assess nutrition knowledge in each area (36). The test time was 25 min. One point was awarded for each question answered correctly, and the correct answer to all 87 questions resulted in a score of 100%. Nutrition knowledge was quantified using the scoring established by Trakman et al. (34).

The questionnaire was expanded to include questions about club membership, age, education, medical conditions, medications taken, dietary advice, source of knowledge about proper nutrition, preparation and type of meals consumed, and adjustment of diet to physical activity. The questionnaire was completed between the 5th and 6th measurements under the supervision of an interviewer during an individual consultation to avoid incomplete answers.

### Statistical analysis

The obtained data were developed using Statistica v.13.3 (Stat Soft Polska) and the R v. 4.0.0 package (2020) under the GNU GPL license (The R Foundation for Statistical Computing).

To present quantitative data, mean values and standard deviations were calculated - X ± S. For qualitative data, percentage notation was used. Qualitative data were expressed as numerical values determined by mathematical methods to make statistical inferences.

Compliance with the normal distribution was checked using the Shapiro-Wilk test. The evaluation of the significance of differences between the means in groups due to the position on the field (goalkeeper, defender, midfielder, and forward) was made using the ANOVA analysis of variance.

For distributions deviating from the normal distribution, their compliance for multiple groups was checked using the Kruskal-Wallis test. For comparisons between the groups, appropriate *post-hoc* tests were performed—Tukey's HSD test for parametric analysis and Dunn's test for non-parametric analysis. Calculations were made for the averaged measurement over the entire period (expressed as the arithmetic mean of the six measurements) and in relation to all individual measurements taken.

To perform a comparative analysis for anthropometric measurements taken at different times, an ANOVA analysis of repeated measurements or a non-parametric Friedman test was performed, depending on the compliance of the distributions with the normal distribution. For comparisons between the groups, appropriate *post-hoc* tests were performed—the HSD Tukey test or the *post-hoc* test for the Friedman test.

An analysis of the level of nutrition knowledge (poor, average, good, and excellent) was also performed in groups, taking into consideration the study group's education (elementary—vocational education and secondary—high education), use of a dietitian (yes and no), and position on the field (goalkeeper, defender, midfielder, and forward). To assess the dependence, the χ^2^ test with variations depending on the sample size or the Fischer test for nxm tables was used.

Spearman's *R*' correlation coefficient with its significance test was used in the correlation analysis of the averaged anthropometric measurements obtained during the study periods in relation to the results obtained in the nutrition knowledge survey.

A value of *p* < 0.05 was used as a criterion for statistical significance.

## Results

A total of 228 body composition measurements were obtained in six different measurements during the spring round of the PKO BP Ekstraklasa 2020/2021 championship, constituting the complete research material. The players were divided into groups according to their function on the field: 5 (13.2%) goalkeepers, 12 (31.6%) defenders, 15 (39.5%) midfielders, and 6 (15.8%) forwards.

Taking education into account, the majority of the respondents had secondary education (*n* = 25; 65.8%). The rest declared elementary education (*n* = 8; 21.1%), vocational education (*n* = 2; 5.3%), and higher education (*n* = 3; 7.9%).

The participants did not report any chronic diseases or ongoing pharmacotherapy (except for one person taking Fostex, a drug used to treat asthma, containing two active substances: beclometasone dipropionate and formoterol fumarate dihydrate).

### Physical characteristics and body composition

The age, physical characteristics (height, body mass, and BMI), and body composition variables (fat-free mass, skeletal muscle mass, and fat mass) in relation to positions on the field are shown in Table 1.

**Table 1:** General physical characteristics and body composition variables—averages (mean ± SD).

**Variable**		**Age (year)**	**Height (cm)**	**Body mass (kg)**	**BMI (kg/m^2^)**	**FFM (kg)**	**SMM (kg)**	**%BF**	**FM (kg)**
**Total (*n* = 38)**		25.89 ± 5.22	182.59 ± 5.45	79.51 ± 7.16	23.82 ± 1.25	71.20 ± 5.77	41.18 ± 3.53	10.16 ± 2.44	8.14 ± 2.39
Field position	F	25.50 ± 5.89	182.50 ± 5.32	79.70 ± 7.55	23.96 ± 1.25	71.65 ± 6.40	41.45 ± 4.06	10.06 ± 1,52	8.02 ± 1.68
	M	24.13 ± 4.69	179.63 ± 4.60	75.10 ± 6.48	23.24 ± 1.24	68.02 ± 5.72	39.08 ± 3.37	9.47 ± 2.28	7.12 ± 1.93
	D	27.50 ± 4.50	183.88 ± 5.11	83.34 ± 5.71	24.63 ± 0.85	74.47 ± 4.50	43.02 ± 2.69	10.68 ± 3.00	8.99 ± 2.82
	G	27.80 ± 7.19	188.50 ± 3.50	83.35 ± 5.60	23.45 ± 1.30	72.37 ± 3.76	42.74 ± 2.30	11.07 ± 2.40	9.30 ± 2.58
	p	0.32	0.01^**^	0.01^**^	0.03^*^	0.01^*^	0.01^*^	0.5	0.14
			**M vs. G**	**M vs. D**	**M vs. D**	**M vs. D**	**M vs. D**		

F, forward; M, midfielder; D, defender; G, goalkeeper; FFM, fat free mass; SMM, skeletal muscle mass; %BF, percentage of body fat; FM, fat mass.

* = p < 0.05;

** = p < 0.01. The bolding is to emphasize statistical significance.

**Figure 2:**
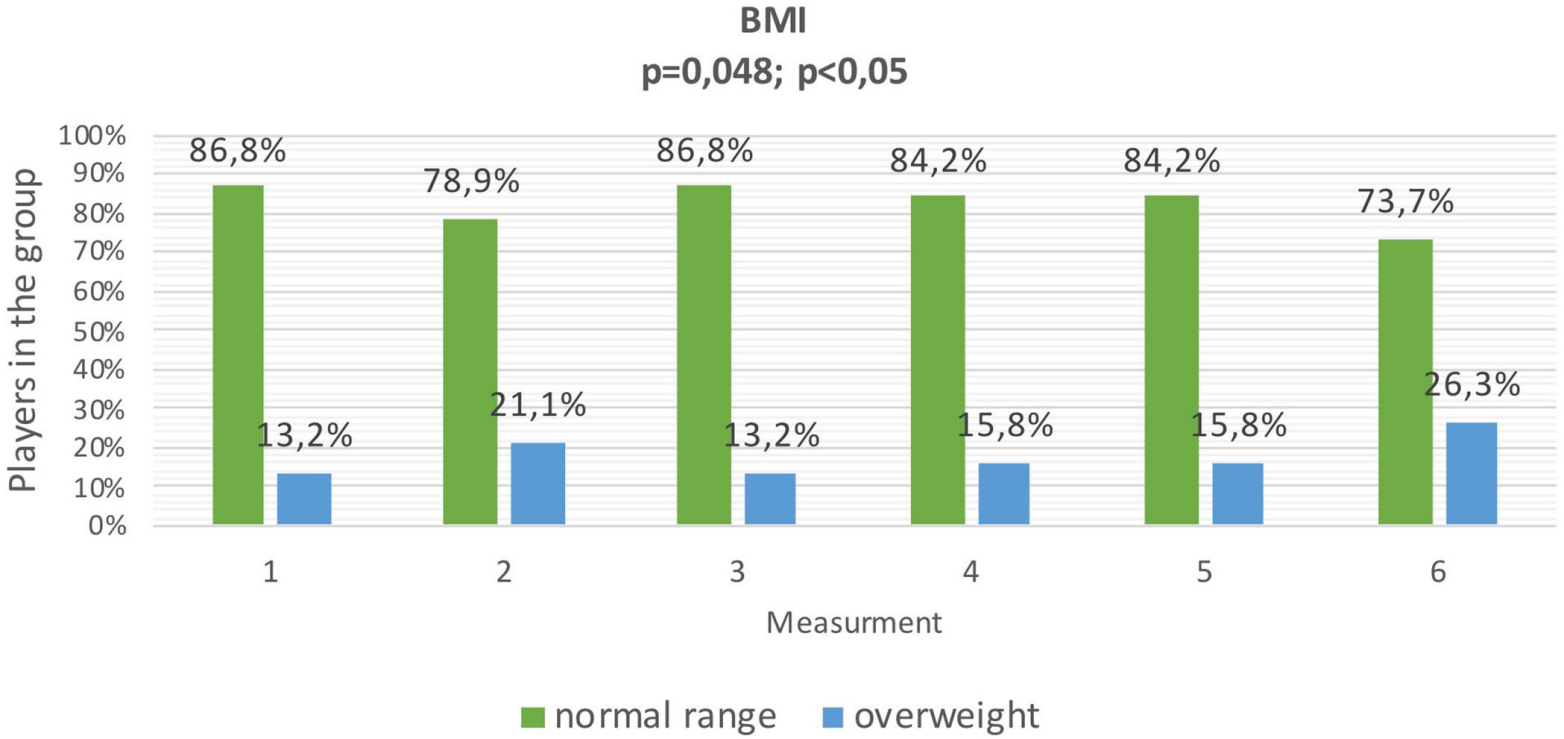
Change in nutritional status of football players assessed by BMI (*n* = 38).

During the measurements, statistically significant differences were found in the prevalence of overweight expressed by BMI. The highest number of athletes with a BMI classified as overweight was found at the 6th measurement, which took place after the end of the transition period, while the lowest number was found at the 1st measurement, which took place at the beginning of the preparatory period, and at the 3rd measurement at the beginning of the competitive period (Figure 2).

**Figure 3:**
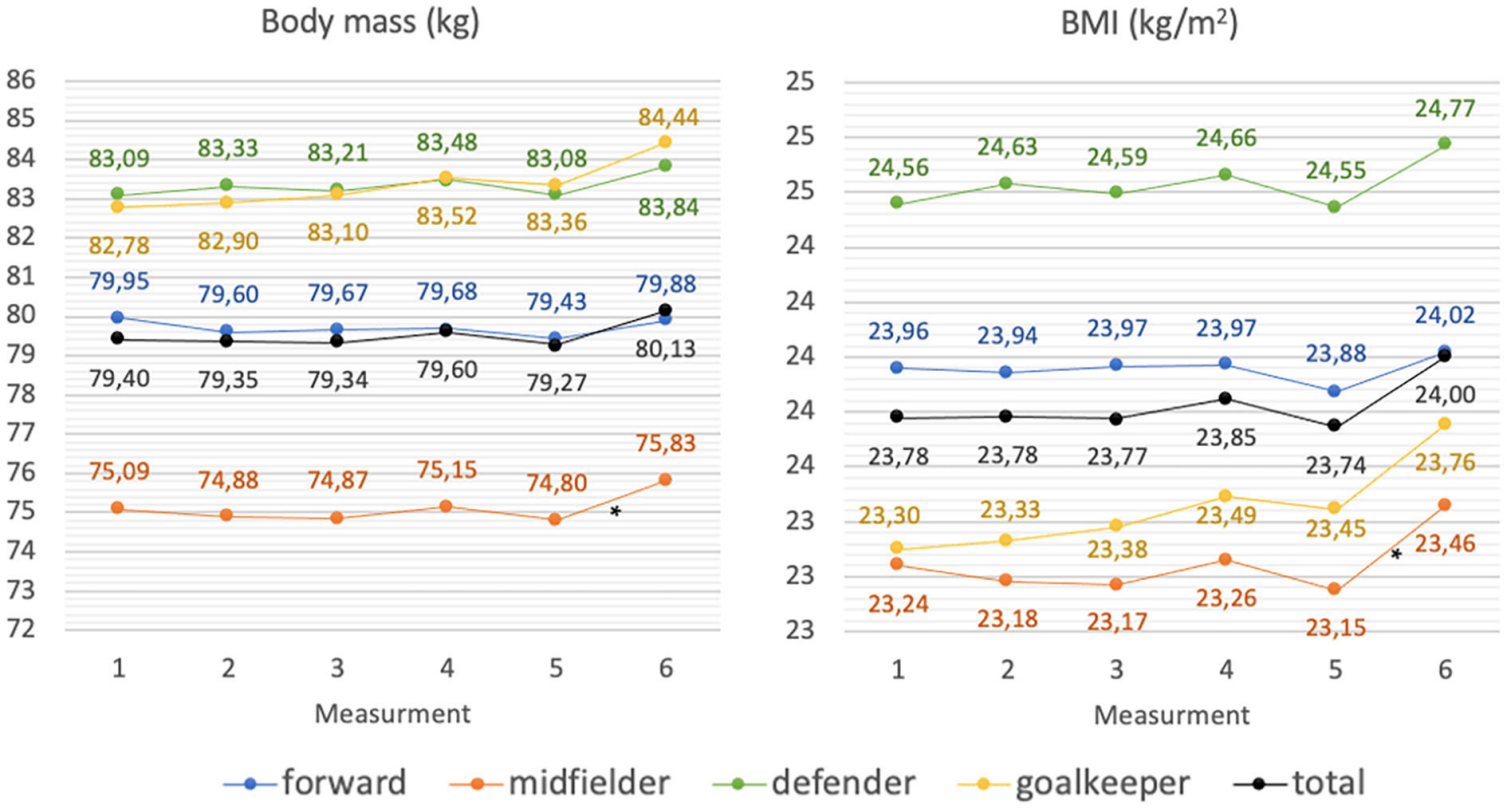
Changes in body mass, and BMI of football players during the spring round of the 2020/2021 football season (average values); *significantly different from (*p* < 0.05).

Statistically significant differences between players performing different functions on the field were found in body mass and BMI measurements. Statistically significantly lower values of analyzed parameters concerned midfielders (*p* < 0.05). In addition, a statistically significant increase in their body mass and BMI values between the 5th and 6th measurements was found (*p* < 0.05). The details are shown in Figure 3.

Statistically significant differences in lean body mass and skeletal muscle mass were found in midfielders, they were characterized by lower content of the above-mentioned body mass components (*p* < 0.05).

**Figure 4:**
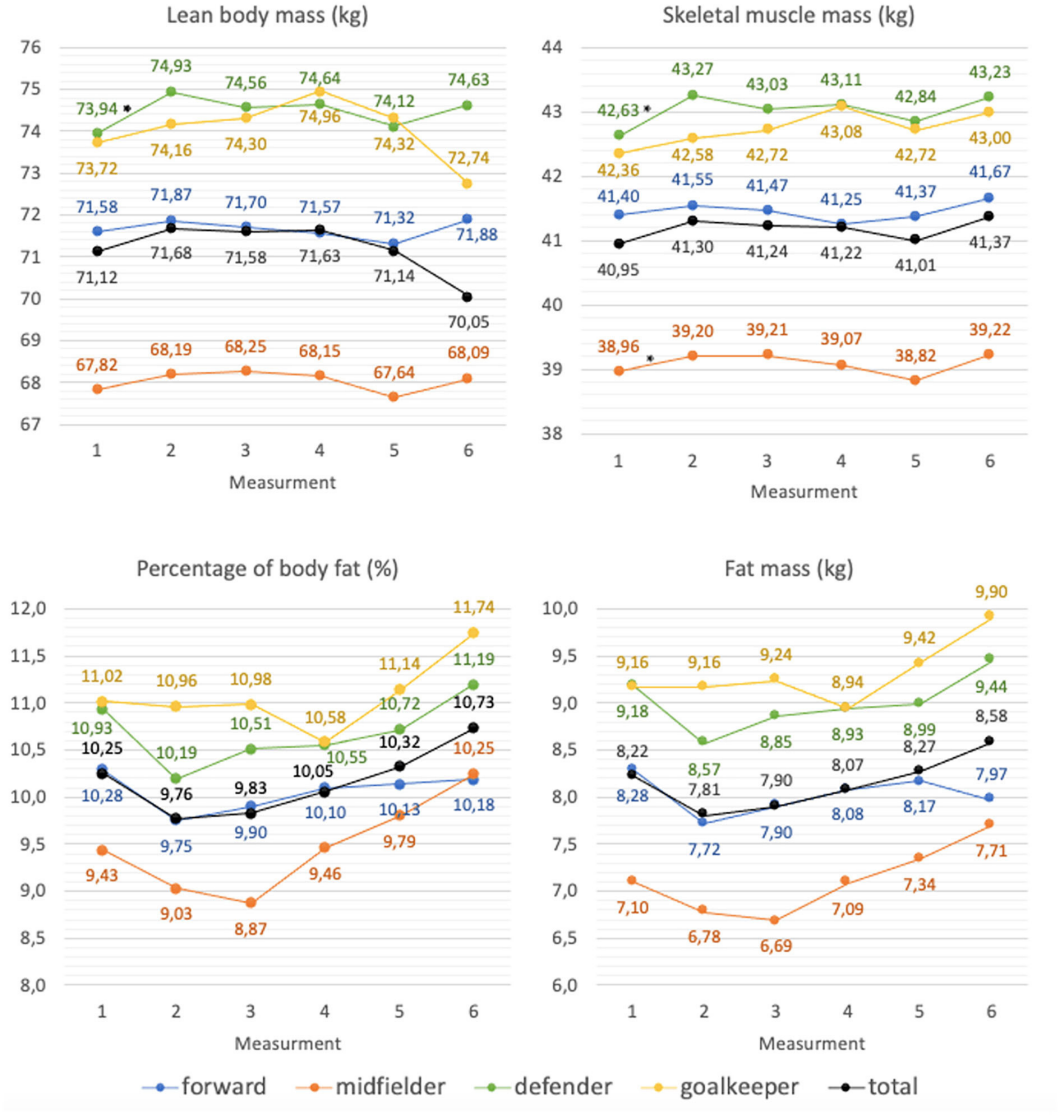
Changes in body composition of football players during the spring round of the 2020/2021 football season (average values); *significantly different from (*p* < 0.05).

An increase in lean body mass content was shown in this group between the 1st and 2nd measurements (*p* < 0.01). The midfielders and defenders groups showed statistically significant increases in skeletal muscle mass content between the 1st and 2nd as well as the 1st and 6th measurements (*p* < 0.05; *p* < 0.01). There were statistically significant increases in the percentage of body fat between the 1st and 6th, the 2nd and 6th, the 3rd and 6th, and the 3rd and 5th measurements in the midfielders group (*p* < 0.05) as well as between the 2nd and 6th measurement in the defender's group (*p* < 0.05). Statistically significant increases in fat mass were shown between the 2nd and 6th, the 3rd and 6th, and the 3rd and 5th measurements in the midfielders group and between the 2nd and 6th measurements in the defenders group (*p* < 0.05; *p* < 0.05). The details are shown in Figure 4.

### Nutrition knowledge

The study groups' knowledge of nutrition was significantly obtained from professional sources. The majority (71%; *N* = 27) of participants received advice from a dietitian, and 44.7% (*N* = 17) of the players at the time of the study. Dietary advice was the primary source of knowledge in this area for 60.5% (*N* = 23). A small group (*N* = 12) of the respondents obtained nutrition knowledge from a coach, as many as 71.1% (*N* = 27) from the Internet, and 34.2% (*N* = 13) from other people. In a question about where meals were eaten, 60.5% (*N* = 23) of the respondents declared eating at home, while 33.3% (*N* = 12) used catering services. Almost half of the athletes prepared their meals (44.7%; *N* = 17), and all players confirmed the adaptation of nutrition to their needs and considered their nutrition to be healthy.

**Figure 5:**
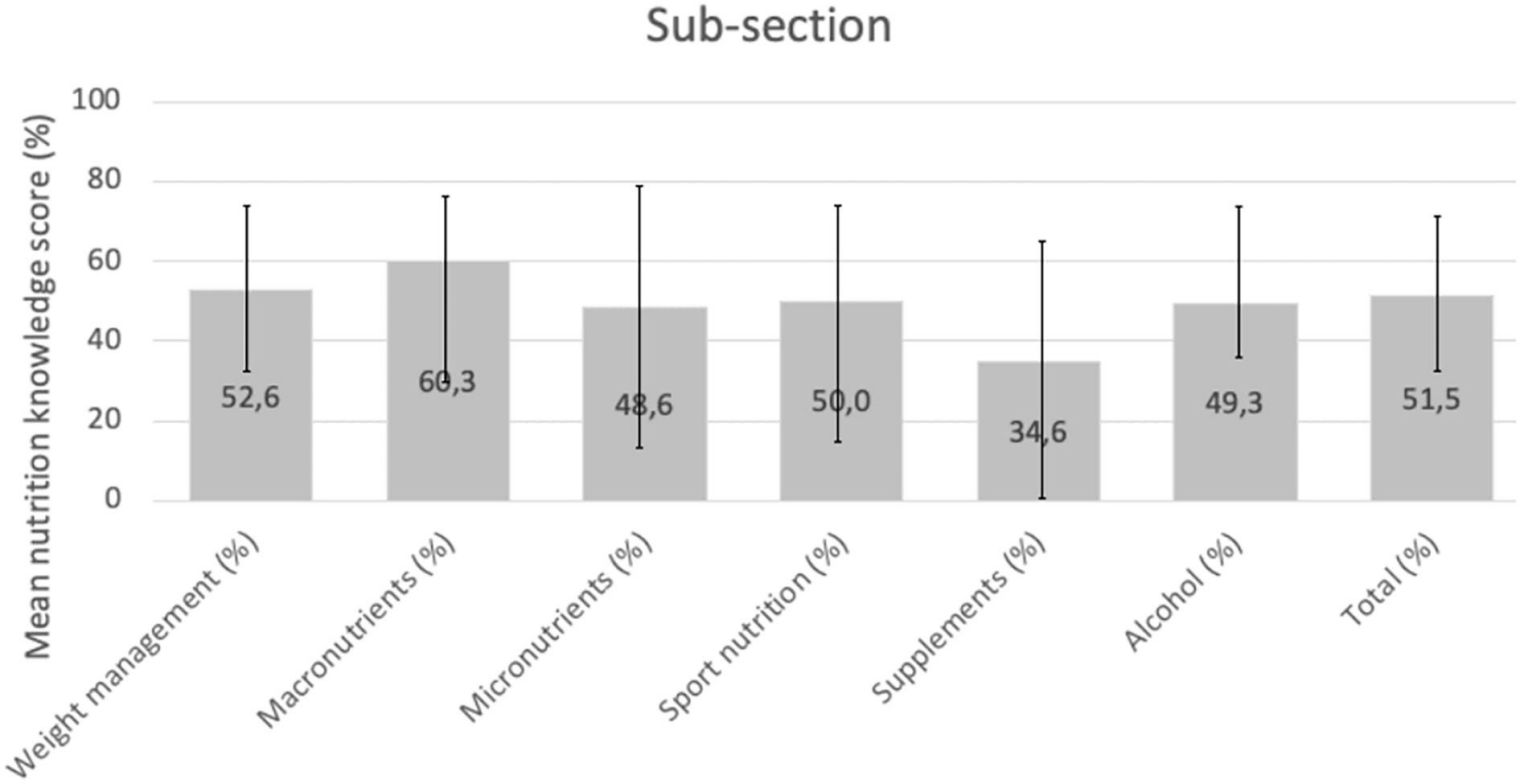
NSKQ scores in football players (*n* = 38). Mean and minimum and maximum percentages are indicated.

Figure 5 shows the average scores and nutrition knowledge divided by categories. On average, the athletes obtained 51.49% of correct answers and their nutrition knowledge was assessed as average. The nutrition knowledge of the studied players regarding micronutrients in the diet, and supplementation was at a poor level, while the nutrition knowledge regarding body weight control, macronutrients in the diet, sports nutrition, and alcohol consumption was at an average level.

**Figure 6:**
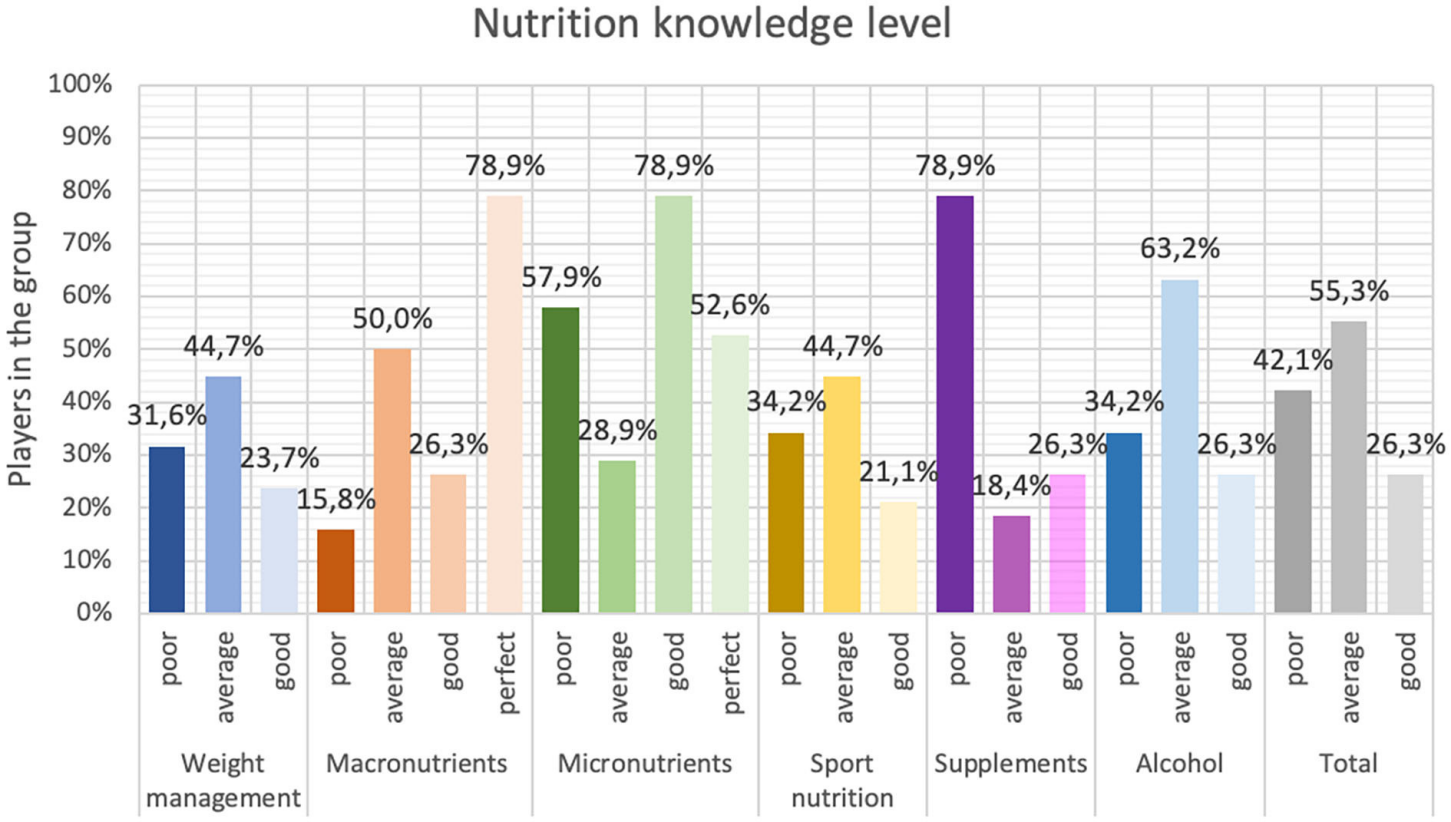
Nutrition knowledge of the football players participating in the study (*n*=38).

Figure 6 shows the percentage distribution of nutritional knowledge of the athletes from each sub-section.

Nutrition knowledge was also verified by taking into account the age of the participants. There was no statistically significant difference in the knowledge in relation to age (*p* > 0.05).

The analysis of nutrition knowledge with regard to the players' level of education was performed similarly. Only in the case of the knowledge of body weight control (*p* = 0.03) and sports nutrition (*p* = 0.03), a higher level of knowledge was found amongst athletes with secondary or higher education.

There was no statistically significant variation in nutrition knowledge amongst athletes under the care of a dietitian (*p* > 0.05).

### Changes in body composition with regard to nutrition knowledge

During the preparatory period, there was a statistically significant negative correlation between knowledge from the subsection of micronutrient content in the diet and the dispersion of body fat content (%) (*r* = −0.36).

In the competitive period, there was a statistically significant negative correlation between knowledge from the subsection of sports nutrition and the dispersion of lean body mass (kg) (*r* = −0.46), and skeletal muscle mass (kg) (*r* = −0.36).

During the transition period, a statistically significant negative correlation between knowledge from the subsection of weight control and the dispersion of body mass (kg) (*r* = −0.47) and BMI values (kg/m^2^) (*r* = −0.48) was identified. Knowledge of the macronutrient subsection is significantly negatively correlated with the dispersion of skeletal muscle mass content (kg) (*r* = −0.33). The results of the analyses are presented in Table 2.

**Table 2:** The strength of correlation of differences between maximum and minimum body composition measurements and scores on the nutrition knowledge assessment expressed by Spearman's R' correlation coefficient.

**Variable**	**Nutrition knowledge sub–section**	**Weight management 0–12 points**	**Macronutrients 0–30 points**	**Micronutrients 0–13 points**	**Sport nutrition 0–12 points**	**Supplements 0–12 points**	**Alcohol 0–8 points**	**Total 0–87 points**
Preparatory period	Body mass (kg)	*r* = −0.14 *p* = 0.42	*r* = 0.08 *p* = 0.65	*r* = −0.09 *p* = 0.60	*r* = −0.18 *p* = 0.28	*r* = 0.01 *p* = 0.94	*r* = −0.19 *p* = 0.26	*r* = −0.11 *p* = 0.52
	BMI (kg/m^2^)	*r* = −0.06 *p* = 0.72	*r* = 0.06 *p* = 0.73	*r* = −0.12 *p* = 0.46	*r* = −0.15 *p* = 0.38	*r* = 0.04 *p* = 0.80	*r* = −0.20 *p* = 0.23	*r* = −0.10 *p* = 0.55
	LBM (kg)	*r* = −0.06 *p* = 0.72	*r* = 0.19 *p* = 0.25	*r* = 0.01 *p* = 0.94	*r* = 0.14 *p* = 0.41	*r* = 0.04 *p* = 0.82	*r* = −0.28 *p* = 0.09	*r* = 0.11 *p* = 0.52
	SSM (kg)	*r* = 0.16 *p* = 0.34	*r* = 0.18 *p* = 0.29	*r* = −0.03 *p* = 0.86	*r* = 0.20 *p* = 0.23	*r* = 0.11 *p* = 0.49	*r* = −0.20 *p* = 0.22	*r* = 0.18 *p* = 0.28
	%BF	*r* = 0.24 *p* = 0.15	*r* = −0.08 *p* = 0.65	***r*** **=** **−0.36** ***p*** **=** 0.03^*^	*r* = 0.00 *p* = 0.99	*r* = 0.11 *p* = 0.51	*r* = −0.17 *p* = 0.30	*r* = −0.11 *p* = 0.53
	FM (kg)	*r* = 0.19 *p* = 0.25	*r* = −0.01 *p* = 0.94	*r* = −0.24 *p* = 0.14	*r* = −0.03 *p* = 0.84	*r* = 0.22 *p* = 0.19	*r* = −0.16 *p* = 0.35	*r* = −0.03 *p* = 0.85
Competitive season	Body mass (kg)	*r* = −0.01 *p* = 0.94	*r* = 0.00 *p* = 0.99	*r* = 0.19 *p* = 0.26	*r* = −0.12 *p* = 0.49	*r* = −0.06 *p* = 0.72	*r* = −0.13 *p* = 0.42	*r* = 0.02 *p* = 0.91
	BMI (kg/m^2^)	*r* = 0.01 *p* = 0.95	*r* = −0.01 *p* = 0.96	*r* = 0.18 *p* = 0.27	*r* = −0.10 *p* = 0.55	*r* = −0.06 *p* = 0.72	*r* = −0.13 *p* = 0.45	*r* = 0.02 *p* = 0.90
	LBM (kg)	*r* = −0.17 *p* = 0.31	*r* = −0.05 *p* = 0.79	*r* = 0.13 *p* = 0.43	***r*** **=** **−0.46** ***p*** **=** 0.004^*^	*r* = −0.13 *p* = 0.43	*r* = −0.23 *p* = 0.16	*r* = −0.20 *p* = 0.23
	SSM (kg)	*r* = −0.13 *p* = 0.45	*r* = −0.13 *p* = 0.45	*r* = 0.06 *p* = 0.73	***r*** **=** **−0.36** ***p*** **=** 0.03^*^	*r* = −0.13 *p* = 0.45	*r* = −0.28 *p* = 0.08	*r* = −0.22 *p* = 0.19
	%BF	*r* = −0.11 *p* = 0.49	*r* = −0.06 *p* = 0.73	*r* = 0.03 *p* = 0.88	*r* = −0.11 *p* = 0.50	*r* = −0.24 *p* = 0.15	*r* = −0.17 *p* = 0.32	*r* = −0.12 *p* = 0.46
	FM (kg)	*r* = −0.09 *p* = 0.6	*r* = −0.04 *p* = 0.82	*r* = 0.08 *p* = 0.61	*r* = −0.08 *p* = 0.65	*r* = −0.20 *p* = 0.22	*r* = −0.08 *p* = 0.63	*r* = −0.06 *p* = 0.70
Transition period	Body mass (kg)	***r*** **=** **−0.47** ***p*** **=** 0.00^***^	*r* = −0.26 *p* = 0.11	*r* = −0.09 *p* = 0.60	*r* = −0.08 *p* = 0.63	*r* = 0.12 *p* = 0.46	*r* = 0.21 *p* = 0.22	*r* = −0.16 *p* = 0.32
	BMI (kg/m^2^)	***r*** **=** **−0.48** ***p*** **=** 0.00^***^	*r* = −0.28 *p* = 0.09	*r* = −0.10 *p* = 0.56	*r* = −0.08 *p* = 0.65	*r* = 0.13 *p* = 0.45	*r* = 0.19 *p* = 0.25	*r* = −0.17 *p* = 0.29
	LBM (kg)	*r* = −0.17 *p* = 0.31	*r* = 0.07 *p* = 0.67	*r* = 0.30 *p* = 0.07	*r* = 0.04 *p* = 0.80	*r* = 0.00 *p* = 1.00	*r* = 0.15 *p* = 0.38	*r* = 0.17 *p* = 0.29
	SSM (kg)	*r* = −0.17 *p* = 0.32	***r*** **=** **−0.33** ***p*** **=** 0.05*	*r* = −0.12 *p* = 0.46	*r* = −0.10 *p* = 0.57	*r* = −0.02 *p* = 0.89	*r* = 0.09 *p* = 0.59	*r* = −0.19 *p* = 0.25
	%BF	*r* = 0.03 *p* = 0.86	*r* = 0.06 *p* = 0.71	*r* = 0.14 *p* = 0.4	*r* = 0.17 *p* = 0.30	*r* = 0.21 *p* = 0.21	***r*** **=** **−0.48** ***p*** **=** 0.00^***^	*r* = 0.29 *p* = 0.08
	FM (kg)	*r* = −0.10 *p* = 0.56	*r* = −0.07 *p* = 0.66	*r* = 0.15 *p* = 0.36	*r* = 0.06 *p* = 0.72	*r* = 0.09 *p* = 0.61	*r* = 0.23 *p* = 0.10	*r* = 0.11 *p* = 0.52

LBM, lean body mass; SMM, skeletal muscle mass; %BF, percentage of body fat; FM, fat mass.

* = p < 0.05;

*** = p < 0.001; r = Spearman's R' correlation. The bolding is to emphasize statistical significance.

## Discussion

Scientific research focuses on the contribution of variables, such as age, sex, eating habits, and training load to maintain the optimal body weight composition of athletes (37, 38). There are few reports indicating how the body composition is shaped during the football periods (6, 19–22).

The changes in body composition are determined by a properly designed training programme and proper nutrition. The training process is planned by the club's training staff, it is adjusted to the possibilities and goals of the team and modified according to individual players' preferences. The coaches, assistants, and physical trainers supervise the correctness of the individual exercises during training, while the activity and participation in games are verified by the coaching staff. Proper nutrition is a highly important aspect of body composition control. Dietitians working in a football club do not usually have regular contact with players, their consultations being limited to occasional meetings. There are about 30 players on the team, and nutrition education for each of them is time-consuming; moreover, the tasks of a dietitian include other responsibilities, e.g., preparing a menu for training camps or assessing of nutritional status of players. Due to the limited contact between the dietitian and the players and the fact that most of the players' meals are consumed outside the club, it is important to assess the players' nutrition knowledge, identify areas that require increased nutrition awareness, and identify the impact of knowledge on changes in body composition during the periods.

Players in various positions on the field have different anthropometric and performance characteristics. Goalkeepers and defenders are usually characterized by higher height and weight than other players (39). Defenders are characterized by higher height and weight compared to midfielders and forwards. Midfielders represent the lowest group of players with the lowest weight (40). The variation in absolute values of lean body mass between players with different roles may be due to differences in body size rather than body composition, so it is important to consider relative values when comparing the body composition of football players (40). Differences between players' body composition are due to the different physiological and metabolic demands of playing football depending on the function on the field (41–43).

In the current research, during the preparatory period, the number of players with a BMI value diagnosed as overweight increased from 13.2% (*N* = 5) at the beginning to 21.1% (*N* = 8) at the end of this period. Analyzing the percentage of body fat over the same period, it was shown that the number of players with a percentage of body fat below the norm has increased. At the beginning of the preparatory period, 51.4% (*N* = 20) of the players had below-normal body fat percentage, while at the end of the preparatory period is applied to 62.2% (*N* = 24) of the respondents. The above-mentioned results confirm that the BMI is not a suitable tool for assessing the body weight of professional athletes, and it is not an appropriate tool for monitoring changes that occur in body weight composition during the preparatory period.

Analyzing the body compositions of the players, an increase in the skeletal muscle mass content was shown on average from 40.95 ± 3.48 kg to 41.3 ± 3.53 kg during the preparatory period. The increase in the content of skeletal muscle mass during this period resulted in a misinterpretation of the BMI index. This study's results do not confirm a significant reduction in the content of adipose tissue in the analyzed period. Results from other studies describe fat reduction with a concomitant increase in lean body mass during the preparation period (20, 44).

Analyzing the authors' own research on the preparatory period, with regards to players' position on the field, a significant increase in lean body mass in the defender's group was shown from 73.94 ± 4.67 kg to 74.93 ± 4.16 kg and skeletal muscle mass from 42.63 ± 2.79 kg to 43.27 ± 2.53 kg. Moreover, in the midfielders group, skeletal muscle content increased from 38.96 ± 3.42 kg to 39.2 ± 3.39 kg.

The preparatory period is followed by the competitive period. Professional football players should adapt their nutrition to the training loads occurring during this period, thus ensuring proper body composition throughout the competitive period (14, 45). In the current study, evaluating the BMI index showed a variable number of overweight athletes over the competitive period. Before the competitive period, 21.1% (*N* = 8) of the athletes were overweight, at the beginning of this period the lowest number of over-weight players was found, only 13.3% (*N* = 5), then this value slightly increased to 15.8% (*N* = 6) in the middle of the period and remained until the end of the period. When analyzing body fat percentage before the start of the competitive period, 62.2% (*N* = 24) of the athletes had below normative body fat percentage, it was the highest number of athletes amongst all measurements. At the beginning of the competitive period, the number of athletes with a below-normal body fat percentage decreased slightly and reached 62.2% (*N* = 24) again by the middle of the competitive period. During the 5th measurement, which was taken at the end of the competitive period, 54.1% (*N* = 21) of the athletes had a below-normal body fat percentage. Similar to the preparatory period, BMI misrepresented changes in body composition during the competitive period in professional athletes. Analysis of body composition during the competitive period in current research showed a decrease in lean body mass from 71.68 ± 5.85 kg to 71.14 ± 5.93 kg and an increase in body fat from 9.76 ± 2.57% to 10.32 ± 2.77%.

In the available literature, researchers have obtained inconclusive results regarding changes in body composition during the competitive period. In a study by Carling et al., lean body mass increased during this period, while fat content decreased (46). In the study by Kultu et al. (47) a reduction in lean body mass and a reduction in body fat were verified. Devlin et al. obtained results consistent with their own, lean body mass was reduced while body fat increased during the competitive period (48). During the preparatory period, training programmes include general training and high-intensity conditioning training, while a significant number of training sessions during the competitive period are related to game tactics, ball possession, and set pieces, which are characterized by less strain. These results suggest that match effort alone is insufficient as an incentive to maintain a consistent body weight of a player (48).

The transition period is characterized by complete cessation or significant reduction of training. Increased body mass resulting from an inappropriate diet affects the performance and efficiency of an athlete. Reduced muscle mass due to lack of training stimulus may result in decreased strength and endurance and thus an increased risk of injury when numerous intensive training units are reintroduced during the preparatory period (14). During the transition period in current research, the number of athletes with a BMI interpreted as overweight increased at the end of the transition period. 26.3% (*N* = 10) of the players were overweight, according to the interpretation of BMI. When analyzing body fat percentage, it was found that most athletes (*N* = 21) had normative body fat at the end of the transition period amongst all measurements analyzed. The transition period is the only period in the analyzed macrocycle when differences in body fat percentage are reflected by the BMI value. It is related to the limitation of physical activity and changes in the composition of the body weight typical of people with low physical activity. This study showed an increase in body weight in the transition period from an average of 79.27 ± 7.44 kg to 80.13 ± 7.47 kg. Similar results apply to the BMI values, which increased from an average of 23.74 ± 1.27 to 24 ± 1.26. Body composition analysis showed a reduction in lean body mass during the transition period and an increase in body fat percentage and body fat mass between the 4th and 6th measurements, i.e., from the second half of the athletic period to the end of the transition period. A study by Reinke et al. showed a reduction in body weight, lean body mass and an increase in body fat in the football players participating in the study (49). Ostojic et al. (50) results are in line with our own.

The authors' study also assessed the nutrition knowledge of professional football athletes; the NSKQ questionnaire was used for the assessment. This study showed that the use of this questionnaire in team sports athletes can be an important tool for screening athletes, requiring additional educational support (34). The athletes in the current study obtained an average of 51.49% of correct answers, and their nutritional awareness was rated as average. The athletes' nutrition knowledge of micronutrients and supplementation was at a poor level, while their nutrition knowledge of weight control, macronutrients, sports nutrition, and alcohol was at an average level.

The NSKQ questionnaire has been used to assess athletes' nutritional awareness by other researchers. A study by Jenner et al. (51) assessed the nutrition knowledge of 46 Australian professional football players. On average, the players obtained 46% of correct answers, and the overall nutrition knowledge of the study group was poor (51). O'Brien et al. analyzed the nutritional awareness of Gaelic football players. On average, the athletes obtained 47.6% of correct answers, and their knowledge was rated as poor (52). A study by Jagim et al. (53) analyzed body composition, nutrition knowledge, and ability to estimate macronutrient and energy requirements of 67 university athletes, including football players. The athletes answered 47.9% of the questions correctly on average, and the knowledge of the study group was rated as poor. In addition, the athletes did not correctly estimate energy and carbohydrate requirements according to their needs. In the above-mentioned study, it was established that the athletes with higher knowledge of the subsection of sports nutrition had lower body fat percentage and body fat mass. Accordingly, athletes with higher levels of sports nutrition knowledge may be better able to adjust macronutrient and energy requirements, thus maintaining desired body composition (53).

In the final stage of the author's study, the contribution of athletes' nutrition knowledge to the maintenance of constant body composition parameters during the preparatory, competitive, and transition periods was determined. The relationship between nutrition knowledge and body composition stability was demonstrated during the competitive period. Higher knowledge of the subsection of sports nutrition corresponded to lower dispersion of lean body mass content (*p* = 0.004), and skeletal muscle mass (*p* = 0.03). The above correlations suggest that athletes with higher nutritional awareness regarding sports nutrition during the competitive period are characterized by less muscle mass loss. During the competitive period, the body composition of the players is disintegrated, the muscle mass is reduced, while the fat tissue content increases, which is a highly unfavorable phenomenon due to the exercise capacity and sport form of athletes. Higher nutritional awareness in individual categories of nutrition knowledge results in the reduction of negative changes in the composition of the body weight during the competitive period. Correlations between nutrition knowledge and body composition stability were demonstrated during the transition period. Higher nutritional awareness of athletes from the weight control subsection corresponded to lower dispersion of body mass (*p* = 0.00) and BMI values (*p* = 0.00). Higher nutrition knowledge on macronutrients was associated with lower dispersion of skeletal muscle mass content (*p* = 0.05), while higher alcohol awareness was associated with lower dispersion of body fat percentage (*p* = 0.00). The described relationships prove that players with higher nutritional awareness of weight control are characterized by a lower increase in body weight and BMI value during the transition period. Athletes who are more knowledgeable about macronutrients in their diets are characterized by lower reductions in muscle mass and footballers with higher awareness of alcohol consumption—with a lower increase in body fat percentage.

The results of this study suggest that football players with higher nutritional knowledge are able to better manage body composition. During the preparatory period, they have better results regarding increasing lean body mass and reducing body fat, which has a positive effect on exercise capacity. During the competitive period, they are characterized by a smaller reduction in lean body mass, which can consequently contribute to a reduced risk of injury. In the transition period, they are characterized by a smaller increase in body mass and better maintain a constant body composition, which affects a quicker return to top athletic form after a period of reduced training intensity. The above results indicate the relevance of the level of nutritional knowledge in football players. Improving the nutrition knowledge of professional football players may provide benefits in terms of better management and maintenance of body composition during particular periods of the macrocycle. No studies were found in the available literature on this issue, so it may be of comparable value.

### Strengths and limitations

The strengths of the study include the fact that despite the popularity of football, there are still few scientific studies analyzing changes in football players' body weight throughout the entire training macrocycle. Typically, body weight composition is assessed over the preparatory, competitive, or transition period, as well as individual measurements, are analyzed, as evidenced by the discussion of the results. Furthermore, in many reports, the study group consists of amateur or semi-professional football players whose training intensity and frequency, match requirements, daily physical activity, as well as salary are different from those of professional players. This study analyzed the results obtained from professional football players of the highest football league in Poland. Assessing the nutrition knowledge of football players is essential to orient education based on the athletes' knowledge insufficiency. Moreover, it can also be useful for screening assessments to identify players who require additional educational support. In the current research, a standardized questionnaire was used for this purpose, which, due to its structure, allows the targeting of educational activities. The results described can provide comparative value with professional athletes practicing other sports. The bibliometric analysis of GoogleScholar indicates that this is the first study analyzing the correlation between nutrition knowledge and changes in body composition in football players (as of 14 May 2022).

One of the limitations of the conducted research is the size of the study group. However, it should be emphasized that many exclusion criteria were defined, one of which was the 14-day exclusion from training related to quarantine or isolation due to COVID-19, which was common in the 2020/2021 football season. The study included players from two football clubs, and the limitation of the study is the lack of representativeness of players from other clubs and other sports disciplines. It is worth emphasizing, however, that each discipline is characterized by a different training macrocycle and a different intensity of matches, which may pose a problem in comparing them reliably. However, it is worth considering such an attempt and such studies are planned by the authors. A control group of non-football players was not included in the study because the type of questions asked would not allow comparisons to be made between such groups.

However, it should be highlighted that prediction equations based on BIA are instrument-dependent, and instrument sensitivities are variable. Therefore, comparisons cannot be made between studies that measure bioimpedance using different technologies (e.g., foot-to-hand or direct segmental measurement in the standing position) or sampling rates. However, it should be pointed out that the authors made every effort to minimize the systematic error in the study (54, 55).

## Conclusion

Based on the results obtained, changes in the composition of the body weight of players were found, regardless of their function on the field in the preparatory, competitive, and transition periods of the PKO BP Ekstraklasa 2020/2021 spring round and their nutrition knowledge was average, according to the adopted classification. BMI did not allow the identification of changes in the composition of the mass (lean and adipose tissue). The applied BIA method more accurately reflected the changes in the body composition of the players in the analyzed periods. The body composition of the athletes, taking into account the spring season periods, underwent significant modifications. During the preparatory period, the mass of skeletal muscles increased, but the mass of fat tissue was stable. During the competitive period, the lean body mass decreased, but the content of fat tissue increased. During the transition period, the content of fat tissue increased in the study group. Correlations were found between elements of nutrition knowledge of athletes and stability of their body composition during all analyzed periods: preparatory, competitive, and transition periods.

## Data availability statement

The raw data supporting the conclusions of this article will be made available by the authors, without undue reservation.

## Ethics statement

The studies involving human participants were reviewed and approved by Bioethics Committee of the Silesian Medical University in Katowice. The patients/participants provided their written informed consent to participate in this study.

## Author contributions

Conceptualization and writing—original draft preparation: WS and EG-N. Methodology: WS, EG-N, and AB-D. Software: MG. Validation: GZ, MK, and SJ-S. Formal analysis: GZ and WS. Investigation: MK. Resources and supervision: OK. Data curation: GZ. Writing—review and editing: AB-D. Visualization: SJ-S. All authors contributed to the article and approved the submitted version.

## Conflict of interest

The authors declare that the research was conducted in the absence of any commercial or financial relationships that could be construed as a potential conflict of interest.

## Publisher's note

All claims expressed in this article are solely those of the authors and do not necessarily represent those of their affiliated organizations, or those of the publisher, the editors and the reviewers. Any product that may be evaluated in this article, or claim that may be made by its manufacturer, is not guaranteed or endorsed by the publisher.

## Footnotes

^1^Football is used to describe association football, the sport most commonly referred to in the English-speaking world as “football” or “soccer”.
